# 

**Published:** 1871-04

**Authors:** 


THREE DOLLARS A YEAR. SINGLE COPIES, 30 CENTS.

VOL. XXVIII. **APRIL, 1871.** NUMBER 4.

THE

### Chicago Medical Journal.

J. ADAMS ALLEN, M.D., LL.D., WALTER HAY, M.D.

EDITORS.

CHICAGO:

W. B. KEEN & COOKE, PUBLISHERS,

113 & 115 STATE STREET.

*The postage on this Journal is 24 cents a year—payable quarterly in advance.*

NOTICE.—Messrs. WILLIAM BALDWIN & CO., No. 177 Broadway, New York, are our General Eastern Agents for THE CHICAGO MEDICAL JOURNAL.

